# A Case of Pancreatic Schwannoma Diagnosed Preoperatively by Endoscopic Ultrasonography-Guided Fine Needle Aspiration and Treated with Laparoscopic Surgery

**DOI:** 10.1089/pancan.2018.0002

**Published:** 2018-05-01

**Authors:** Kazuao Hayashi, Atsunori Tsuchiya, Satoshi Ikarashi, Kazuyasu Takizawa, Shuji Terai

**Affiliations:** ^1^ Division of Gastroenterology and Hepatology, Graduate School of Medical and Dental Sciences, Niigata University, Niigata, Japan.; ^2^ Division of Digestive and General Surgery, Graduate School of Medical and Dental Sciences, Niigata University, Niigata, Japan.

**Keywords:** schwannoma, calcification, endoscopic ultrasonography-guided fine needle aspiration, S-100, laparoscopic surgery

## Abstract

**Background:** Pancreatic tumors are often difficult to diagnose in atypical cases, and a pancreatic schwannoma is very rare. We present a case of pancreatic schwannoma with calcification diagnosed preoperatively by endoscopic ultrasonography (EUS)-guided fine needle aspiration (FNA) and treated with laparoscopic distal pancreatectomy.

**Presentation:** A 72-year-old-woman was admitted to our hospital due to a 6 × 4.5 cm large tumor in the pancreatic tail. Imaging modalities revealed that the tumor was hypovascular and gradually enhanced with calcification, but was without cystic lesions. EUS revealed the tumor had a clear boundary with a low echoic mass. EUS-FNA was performed and spindle-shaped cells that were immunopositive for S-100 and negative for c-kit, CD34, and desmin were detected, resulting in a diagnosis of schwannoma. Laparoscopic distal pancreatectomy with splenectomy was safely performed without recurrence for a year.

**Conclusions:** Schwannoma is very rare; however, characteristics of the tumor, such as calcification, can help the diagnosis and, if possible, EUS-FNA should be performed for an appropriate treatment decision.

## Introduction and Background

Pancreatic tumor is often difficult to diagnose in atypical cases. Pancreatic schwannoma is a very rare tumor; <50 cases of pancreatic schwannoma have been described in the English literature for the past 30 years.^1^ Here we present a case of pancreatic schwannoma with calcification and without cystic lesions that was diagnosed preoperatively by endoscopic ultrasonography (EUS)-guided fine needle aspiration (FNA) and treated with laparoscopic surgery.

## Presentation of Case

**FIG. 1.**
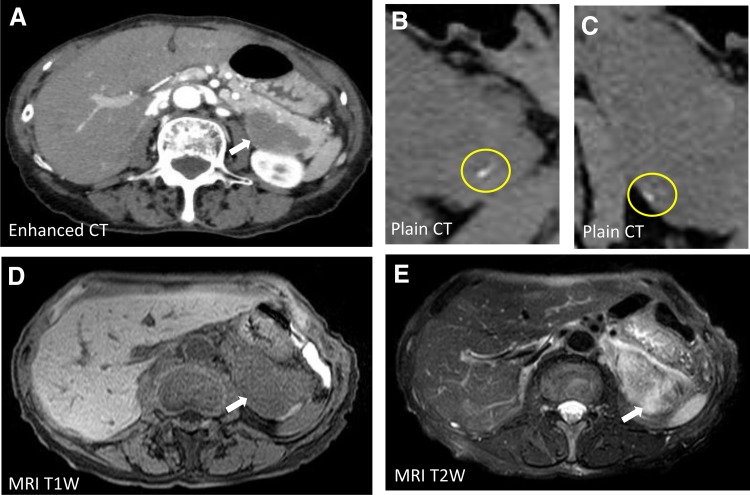
CT and MRI. **(A)** CT revealed a 6-cm hypovascular tumor in the pancreatic tail, indicated by *white arrow*. **(B, C)** Calcification was detected at the edge of the tumor. *Yellow circles* indicate calcification. **(D, E)** MRI showed hypointensity of the tumor on T1-weighted images **(D)**, and hyperintensity on T2-weighted images **(E)**; tumor indicated by *white arrows*. CT, computed tomography; MRI, magnetic resonance imaging.

**FIG. 2.**
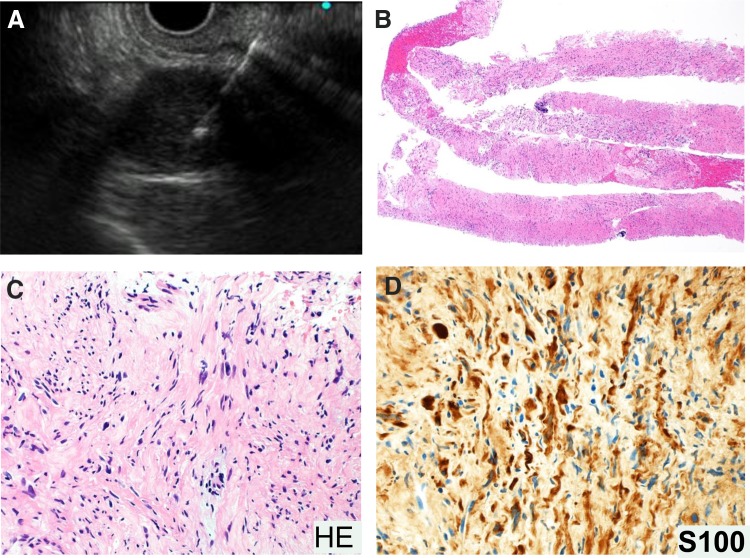
EUS-FNA and histological analysis. **(A)** EUS-FNA was performed against the clear boundary of the low echoic mass. **(B–D)** Spindle-shaped cells were detected by hematoxylin-eosin staining **(B**: original magnification ×40, and **C**: original magnification ×200**)**; these cells were positive for S-100 **(D**: original magnification ×200**)**. EUS-FNA, endoscopic ultrasonography-guided fine needle aspiration; HE, hematoxylin and eosin stain.

A 72-year-old-woman was admitted to our hospital with a 6 × 4.5 cm huge tumor in which an artery was penetrating in the pancreatic tail (Fig. 1A, white arrows). She had a history of hypertension, spinal canal stenosis, and inappropriate antidiuretic hormone syndrome. She had no abdominal symptoms; however, survey for malignancy helped detect the tumor incidentally. Laboratory data revealed a low white blood cell count of 2440/μL (normal range [NR]: 3300–8600) and low sodium level (133 mEq/dL, NR: 138–145). The following tumor markers were all within the respective NRs: carcinoembryonic antigen (4.1 ng/mL, NR: ≦5.8); carbohydrate antigen 19–9 (12 U/mL, NR: ≦37); duke pancreatic antigen type 2 (<25 U/mL, NR: ≦150); and s-pancreas antigen-1 (8.5 U/mL, NR: ≦30). Contrast-enhanced computed tomography revealed calcification at the edge of the tumor (Fig. 1B, C), no cystic lesion, and gradual enhancement. Magnetic resonance imaging revealed hypointensity of the tumor on T1-weighted images (Fig. 1D) and hyperintensity on T2-weighted images (Fig. 1E). EUS revealed the tumor had a clear boundary with a low echoic mass. We suspected that it was a neuroendocrine tumor, acinar cell carcinoma, solitary-pseudopapillary neoplasm, schwannoma, or anaplastic ductal carcinoma based on the calcification, gradual enhancement, and expansive growth. Therefore, EUS-FNA was performed (Fig. 2A), and spindle-shaped cells (Fig. 2B, C) that were immunopositive for S-100 (Fig. 2D) and negative for c-kit, CD34, and desmin were detected. Therefore, the final diagnosis was schwannoma. Laparoscopic distal pancreatectomy with splenectomy was performed and the patient is currently well without recurrence for 1 year.

## Discussion

Schwannomas are neurogenic neoplasms derived from Schwann cells of the peripheral nerve sheaths. Schwannoma is often detected in the head and neck, extremities, mediastinum, and retroperitoneum; however, it is very rare in the pancreas.^2^ Schwannoma often contains a solid component with areas of degenerative changes such as a cyst, calcification, hemorrhage, and hyalinization.^3^ In this case, calcification at the edge of the tumor was detected; therefore, when calcification is detected in the tumor, a diagnosis of schwannoma should be considered. Precise preoperative diagnosis of a pancreatic schwannoma is challenging because the clinical symptoms and radiological characteristics of schwannomas are nonspecific. Schwannomas have characteristic spindle cells with myxoid stroma, and are immunopositive for S-100 (differential diagnosis; Table 1).^1,2,5–8^ The majority of schwannomas are benign; however, malignant cases that often have cystic formation and/or large tumor size (5 of 47 cases; 10.6%) are sometimes reported.^2^ An appropriate diagnosis regarding the potential for malignancy has to be performed before a treatment decision can be made. In our case, the tumor was relatively huge and hypovascular, thus we cannot exclude the malignant potential of the tumor; however, EUS-FNA definitively diagnosed the schwannoma and estimated the malignant potential, after which a minimally invasive laparoscopic distal pancreatectomy with splenectomy was performed without recurrence. If the biopsy revealed that the primary tumor was a malignant schwannoma or any other malignant tumor, we selected laparotomy as the surgical procedure. EUS-FNA can often obtain only very small specimens, and there have been some reports stating that only a small proportion of histologically proven schwannomas can be diagnosed correctly using EUS-FNA, which imposes a massive limitation.^4^ However, in this case, EUS-FNA did provide an accurate diagnosis, resulting in an appropriate treatment. Development of a new EUS-FNA device would be possible to achieve more accurate diagnosis.

**Table 1.** **Differential Diagnosis of Schwannoma**

	Schwannoma	NET	ACC	SPN
Imaging technique				
Enhanced CT	Well-defined hypovascular tumor with delayed enhancement	Well-defined hypovascular tumor	Well-defined iso-hypovascular tumor	Well-defined hypovascular tumor
MRI	T1 low/T2 high	T1 low/T2 high	T1 low/T2 iso-high	T1/T2: low-high-mix
Pathology	Spindle-shaped cells with myxoid stroma	Trabecular/pseudorosette structure	Acinar structure	Pseudopapillary structure
IHC				
Positive makers	S-100, vimentin	CD56, chromogranin A, synaptophysin	Trypsin, BCL10	*β*-catenin, vimentin

NET, neuroendocrine tumor; ACC, acinar cell carcinoma; SPN, solitary-pseudopapillary neoplasm; CT, computed tomography; IHC, immunohistochemistry; MRI, magnetic resonance imaging.

## Conclusion

Schwannoma is a very rare tumor; however, characteristics identified in imaging studies, such as calcification, hemorrhage, and hyalinization, can help make an accurate diagnosis. Furthermore, the potential for malignancy needs to be estimated to provide appropriate treatment. Therefore, if possible, EUS-FNA should be performed before a treatment decision is made.

## Abbreviations Used

CT: computed tomography
EUS: endoscopic ultrasonography
FNA: fine needle aspiration
MRI: magnetic resonance imaging
